# A Deep Convolutional Neural Network Inspired by Auditory Perception for Underwater Acoustic Target Recognition

**DOI:** 10.3390/s19051104

**Published:** 2019-03-04

**Authors:** Honghui Yang, Junhao Li, Sheng Shen, Guanghui Xu

**Affiliations:** School of Marine Science and Technology, Northwestern Polytechnical University, Xi’an 710072, China; ljhhjl@mail.nwpu.edu.cn (J.L.); shensheng@mail.nwpu.edu.cn (S.S.); hsugh@mail.nwpu.edu.cn (G.X.)

**Keywords:** underwater acoustic target recognition, ship-radiated noise, deep learning, brain-inspired, auditory perception inspired, filter learning

## Abstract

Underwater acoustic target recognition (UATR) using ship-radiated noise faces big challenges due to the complex marine environment. In this paper, inspired by neural mechanisms of auditory perception, a new end-to-end deep neural network named auditory perception inspired Deep Convolutional Neural Network (ADCNN) is proposed for UATR. In the ADCNN model, inspired by the frequency component perception neural mechanism, a bank of multi-scale deep convolution filters are designed to decompose raw time domain signal into signals with different frequency components. Inspired by the plasticity neural mechanism, the parameters of the deep convolution filters are initialized randomly, and the is n learned and optimized for UATR. The n, max-pooling layers and fully connected layers extract features from each decomposed signal. Finally, in fusion layers, features from each decomposed signal are merged and deep feature representations are extracted to classify underwater acoustic targets. The ADCNN model simulates the deep acoustic information processing structure of the auditory system. Experimental results show that the proposed model can decompose, model and classify ship-radiated noise signals efficiently. It achieves a classification accuracy of 81.96%, which is the highest in the contrast experiments. The experimental results show that auditory perception inspired deep learning method has encouraging potential to improve the classification performance of UATR.

## 1. Introduction

Underwater acoustic target recognition using ship-radiated noise faces big challenge due to the complexity of the ocean environment and the application of acoustic stealth technology. Underwater acoustic target recognition based on machine learning methods is the research emphasis in the area of underwater acoustic signal processing. Traditional underwater acoustic target recognition methods via ship-radiated noise use hand designed features and shallow classifiers to classify ship types. The traditional underwater acoustic target recognition methods can be divided into the following aspects: (1) Feature extraction; (2) Feature selection; (3) Classifier design. The hand designed features of ship-radiated noise include waveform features [1], spectrum features [2], wavelet features [3] and so on. The se hand designed features, which depend on expert knowledge and prior knowledge, have weak generalization ability. Although the noise features or redundant features can be removed by feature selection methods [4], the inherent generalization ability problem of these features still cannot be solved radically. The shallow classifiers, such as support vector machine (SVM) [5] and shallow neural classifier [6], have weak fitting capacity and weak generalization ability while processing complex and large number of samples. Thus, the underwater acoustic target recognition still mainly relies on well-trained sonar man.

Neuroscientists discovered that the human auditory system has unique superiority in term of sound recognition. This is mainly due to the strong ability of human brain in perception, reasoning, induction, learning and other aspects. The refore, inspired by human neural structure and information processing mechanisms of brain, deep neural networks (DNNs) have been proposed to process information and make decisions in a brain-like manner.

Recently, several underwater acoustic target recognition methods based on deep learning have been proposed. SAE-softmax model [7] was proposed to extract deep feature from spectrogram of ship-radiated noise. Yue [8] utilized deep belief network (DBN) and convolutional neural network (CNN) to extract deep feature from spectrum of ship-radiated noise. We [9] proposed a competitive deep belief network (CDBN) by combining competitive learning mechanism and DBN. The competitive learning mechanism could enhance discriminating information of deep features, and the CDBN achieved better recognition result than conventional DBN when processing the frequency information of ship-radiated noise. All these deep learning methods could achieve better or equivalent recognition results compared to traditional underwater acoustic target recognition methods. The researches mentioned above borrowed some ideas from the neural structure or the information processing mechanisms of brain to some extent, but the auditory information processing mechanisms of auditory system were not considered enough. To better recognize underwater acoustic targets, it is considerate to make models realize the functions that are more similar with the auditory system [10].

For human, the sound perceiving and recognition is accomplished by the auditory system including auditory periphery and auditory center [11]. The process of auditory perception is roughly as follows: Firstly, the cochlea receives the acoustic signals and produces nerve impulses. The n, the frequency, intensity and other information of the sound are transmitted by nerve impulse through the auditory nerve to the auditory center. Finally, the information is merged and identified at auditory cortex. With the development of neuroscience, more neural mechanisms of auditory perception have been revealed. The researchers discovered that some structures of deep auditory system have the ability of frequency decomposition in different range and resolution, for example, cochlea [12,13], auditory midbrain [14], primary auditory cortex, secondary auditory cortex [15,16,17] etc. When performing frequency analysis, primary auditory cortex can decompose the complex acoustic signal into different frequency components by nerve cells with multi-scale frequency receptive fields [15]. In addition, different frequency components in acoustic signal can activate different areas in auditory system. Complex signal with multiple frequency components can activate more areas, which are widely distributed in primary auditory cortex and secondary auditory cortex [16,17]. Other researchers devoted to the study of brain plasticity, which means the brain could adjust its structure and function to meet the needs of learning tasks [18]. In the auditory center, the frequency sensing related areas in the auditory cortex, auditory midbrain and other structures can adjust the frequency receptive fields and the optimal frequency to complete the learning tasks [19,20]. The discoveries of these researches on auditory system suggest that: (1) the acoustic signals in time domain are decomposed in frequency components in auditory system; (2) the information of different frequency components is perceived in different regions of auditory system; (3) The brain synthesizes information from all of the regions to analyze and classify the acoustic signals. Furthermore, research on auditory cortical plasticity has proved that the adult brain can be reshaped under the right circumstances. The function and even structure of the auditory system can be changed as a result of auditory experience [21].

Inspired by the achievements of neuroscience mentioned above, in this paper, we present an end-to-end deep neural network, named ADCNN, for the underwater acoustic target recognition. The proposed ADCNN model is composed of a series of deep filter sub-networks, fusion layers and decision layer. In the deep filter sub-networks, firstly the deep convolution filters with varying convolution kernel width decompose raw time domain ship-radiated noise signal into signals with different frequency components. Next, max-pooling layers and fully connected layers are utilized to extract features from each decomposed signal. Finally, in the fusion layers, the deep features are merged by the merging layer, and then the feature representations that are more correlative with ship categories are obtained by followed full connected layers to fit the input of the decision layer for underwater acoustic target recognition, which matches information merging and acoustic signal recognition function at auditory cortex.

The contribution of this paper is briefly summarized as follows:

1. In the proposed model, a deep filter sub-network, which is composed of deep convolution filters, max-pooling and several full connected layers, is presented to simulate the deep acoustic information extraction structure of auditory system.
2. Inspired by the frequency component perception neural mechanism, the complex frequency components of ship-radiated noise are decomposed and modeled by a bank of multi-scale deep filter sub-networks.
3. Inspired by the plasticity neural mechanism, the parameters of the multi-scale deep filter sub-networks are learned from the raw time domain ship-radiated noise signals.
4. The experimental results demonstrate that the proposed ADCNN model is effective for underwater acoustic target recognition. It can decompose, model and classify ship-radiated noise signal efficiently, and achieve better classification performance than the compared methods.

This paper is organized as follows. Section 2 gives an overview of the proposed ADCNN. Section 3 describes details of the proposed method. Section 4 describes experimental data. Experimental setup and results are presented and discussed in Section 5. The conclusion is discussed in Section 6.

## 2. Auditory Perception Inspired Deep Convolutional Neural Network for UATR

### 2.1. The Neural Mechanisms of Auditory Perception

Sonar men identify objects of interest from complex marine environments depending on the powerful information processing ability of the auditory system. According to the discoveries in neuroscience, the deep acoustic information extraction structure and some referable neural mechanisms of the auditory perception are summarized.

The deep acoustic information extraction structure of auditory system means auditory system is a multi-level system mainly including cochlea, auditory midbrain, auditory thalamus and auditory cortex. When processing acoustic signal, different frequency components of acoustic signal can be decomposed in the auditory pathway from cochlea to midbrain to auditory cortex.

The neural mechanisms summarized in this paper include frequency component perception neural mechanism and plasticity neural mechanism. For the frequency component perception neural mechanism, the decomposed information with different frequency components can be perceived by different areas which exist in cochlea, auditory midbrain, primary auditory cortex, secondary auditory cortex etc. And the decomposed information with similar frequency components can activate relatively fixed area. As to the plasticity neural mechanism, driven by different acoustic stimulation in different learning tasks or environments, the auditory system could continuously adjust its auditory perception ability to meet the need of the learning task. The plasticity property of auditory system runs through the whole process of auditory perception.

### 2.2. The Architecture of ADCNN for UATR

**Figure 1:**
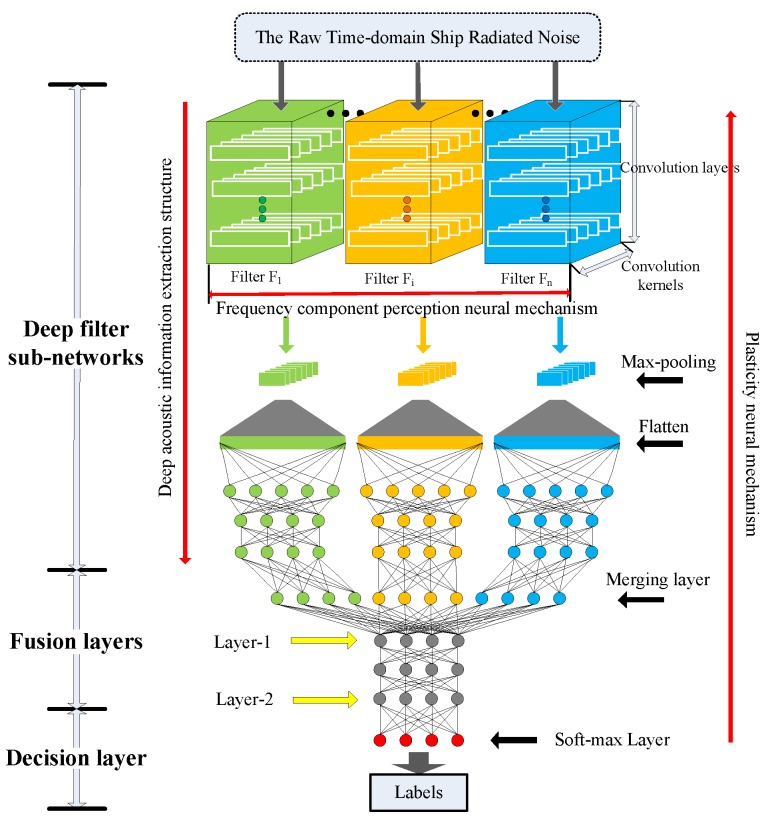
The architecture of ADCNN.

Inspired by auditory perception, ADCNN is proposed for ship-radiated noise modeling and ship type classifying. The proposed model includes a series of deep filter sub-networks $S=\{S_{1},S_{2},\ldots ,S_{k}\}$, fusion layers and decision layer. The architecture of the proposed model is shown in Figure 1.

The deep filter sub-networks realize the frequency decomposition of the input signals and feature extraction of the decomposed signals. Inspired by the deep acoustic information extraction structure of auditory system, a deep filter sub-network is designed in the proposed model. Each deep filter sub-network $S_{i}$ is a multi-layer convolutional neural network followed by max-pooling layer and several full connected layers. The multi-layer convolutional neural network in each deep filter sub-network is designed as deep convolution filter $F_{i}$ to extract information of frequency component in raw acoustic data. The amplitude features of extracted frequency component information are obtained in max-pooling layer. The n features are further extracted in full connected layers. For all deep filter sub-networks, inspired by the frequency component perception neural mechanism, deep convolution filters $F=\{F_{1},F_{2},\ldots ,F_{k}\}$ with varying convolution filter width is presented to decompose the complex raw acoustic data into different frequency components. All deep filter sub-networks are optimized in parallel.

The fusion layers realize feature fusion. The fusion layers are composed of merging layer and some full connected layers. In the merging layer, the outputs of all deep filter sub-networks are merged to comprehensively analyze. The n the merged features are passed to some full connected layers to fit the input of the decision layer.

In the decision layer, the softmax layer is utilized to obtain a prediction probability over every ship type for each sample. The parameters in the whole network are initialized randomly, and then driven by raw ship-radiated noise signals correlatively with ship categories, learned and optimized during the whole training process. This optimization mechanism reflects the fact that the auditory system has the plasticity neural mechanism.

With such architecture, proposed model can realize ship-radiated noise decomposition, feature extraction and classification for underwater acoustic target recognition task.

## 3. Detailed Implementation of ADCNN for UATR

### 3.1. Learned Deep Filter Sub-Network

In the proposed model, each deep filter sub-network is composed of deep convolution filter, max-pooling layer and many full connected layers. The deep convolution filter is a multi-layer CNN. CNN is a kind of artificial neural network which performs a series of convolutions over input signal. Convolution operation in the CNN is equivalent to time domain convolution in conventional filter [22]. In this paper, multi-layer CNN in each deep filter sub-network is designed to realize filtering function, so we define that as a deep convolution filter.

In the deep filter sub-network $S_{i}$, deep convolution filter $F_{i}$ is composed of *m* convolution layers. The outputs of layer l−1(l=2,3,…,m) are convolved with learnable kernel $k_{g,j}^{l}$ and put through the activation function to form the output feature maps. Each output feature map $x_{j}^{l}$ may combine convolutions with multiple input feature maps. Each output feature map is given an additive bias $b_{j}^{l}$.

(1) $$x_{j}^{l}=f\left(\sum_{g\in q^{l-1}}x_{g}^{l-1}*k_{g,j}^{l}+b_{j}^{l}\right)$$

where *g* represents a selection of input feature maps.

By repeating the above procedure layer by layer, the constructed multi-layer CNN could extract more abstract features in a deep architecture. Units in the deeper layers can be indirectly connected to all or most of the signal. The receptive field of the units in the deeper layers of a deep filter sub-network is larger than that in the shallow layers [23]. The parameters of the deep convolution filters are initialized randomly and learned from acquired ship-radiated noise. In this way, the learned filters are more suitable for the underwater acoustic target recognition task.

After deep filtering, the max-pooling operation is performed on the features extracted by the deep convolution filter to extract amplitude features. The output of max-pooling layer is passed to several full connected layers for further feature extraction. The deep structure of filter sub-network reflects the deep acoustic information extraction structure of auditory system.

### 3.2. Ship Radiated Noise Signal Decomposition with a Bank of Multi-Scale Deep Filter Sub-Networks

In the auditory system, the acoustic signal with multi-frequency components can be decomposed into different frequency components and different frequency components of acoustic signal can activate different areas [15,16,17]. Driven by different acoustic stimulation, the frequency related auditory cortex regions, auditory midbrain and other tissues could adjust the frequency receptive fields to better complete the auditory tasks [19,20]. Moreover, while building deep neural networks, Arora [24] suggests a layer-by-layer construction in which one should analyze the correlation statistics of the last layer and cluster them into groups of units with high correlation.

Inspired by frequency component perception neural mechanism, we construct *k* deep filter sub-networks $S=\{S_{1},S_{2},\ldots ,S_{k}\}$ with the filters $\left\{F_{1},F_{2},\ldots ,F_{k}\right\}$, and the convolution kernel width in filter $F_{i}$ is different from that in filter $F_{j}$. Convolution kernels with the same width are wrapped in one filter sub-network. Convolution kernels in one filter sub-network are more correlated with each other. As for ship-radiated noise, the radiated noise signal energy of different ship types concentrates in different frequency band, and signal components with similar frequency are more correlative with each other. So when signal s(n) passes through the filters with multi-scale convolution kernels, the outputs of the filters may have different frequency components. Driven by the time domain signals of ship-radiated noise, the frequency decomposition ability of the deep convolution filters is learnable and adjustable. In addition, the larger convolution kernels can contain longer wavelengths, which means the frequencies of the components is lower, and vice versa.

The process can be expressed in two aspects: Multi-scale convolution kernels can be viewed as multi-scale receptive field, which could analyze multi-scale temporal structure in ship-radiated noise signals. The outputs of deep filter sub-networks can be viewed as densely distributed feature subset, in which highly correlated features are grouped together, thus ship-radiated noise can be decomposed into components with different frequency. In a word, in the deep filter sub-networks of the proposed model, filters with convolution kernels of different scales are set up to obtain different frequency components in different filter sub-networks, so as to simulate the response of different regions of auditory system to specific frequency.

In the fusion layers, the outputs of deep filter sub-networks are merged in merging layer. The n deeper feature representations correlative with ship categories are extracted by several full connected layers. Finally, the se deep feature representations are fit the input of the decision layer. The decision layer makes the final prediction of ship type for input signal using softmax function.

### 3.3. The Plasticity of ADCNN Model for Underwater Acoustic Target Recognition

Some neuroscience researchers have found that the brain could change its structure and function to match the needs of learning tasks [15]. Driven by different acoustic stimulation, the frequency related auditory cortex regions, auditory midbrain and other tissues could adjust the frequency receptive fields to better complete the auditory tasks [16,17].

In the proposed model, deep filter sub-networks can realize the frequency perception and decomposition function of the auditory system to some extent. Driven by the time domain signals of ship-radiated noise, all parameters of deep filter sub-networks are learned from real data. The frequency decomposition and perception ability of the deep filter sub-networks is also learnable and adjustable. This plasticity of frequency perception and decomposition can reflect the plasticity of brain. The whole ADCNN model is optimized with RMSProp algorithm. The pseudo-code of RMSProp is shown in Table 1. The ADCNN is trained to learn discriminative features from frequency distribution of different ship types and to match the task of ship type classification. The optimization of ADCNN in end to end manner reflects the plasticity neural mechanism of auditory perception.

## 4. Experimental Dataset

The ship-radiated noise is acquired by Ocean Networks Canada observation. The signals are recorded by an Ocean Sonics icListen AF hydrophone placed at Latitude $49.080811^{\circ }$, Longitude $-123.3390596^{\circ }$ and 144 m below sea level. The sampling frequency is 32 kHz. The labeling of the signals is performed by combining these recordings with automatic identification system (AIS). The signals generated when only one ship appearing within an area of 2 km radius of the hydrophone are recorded.

**Figure 2:**
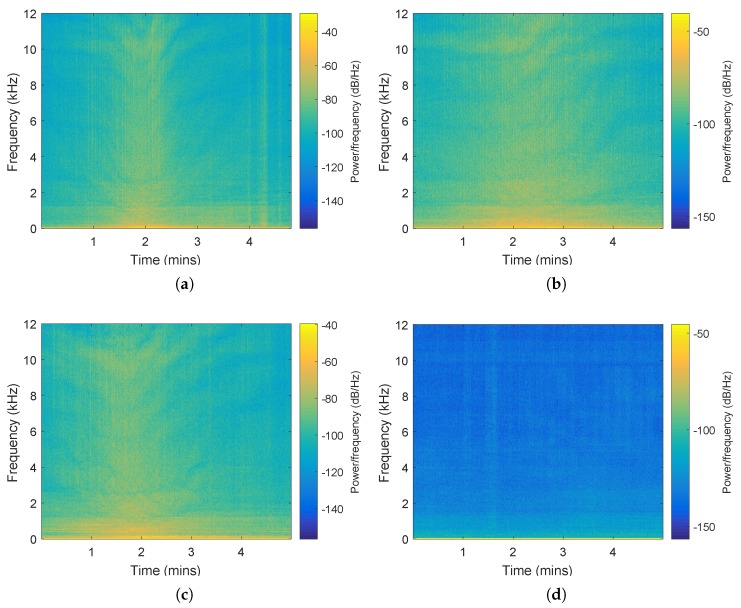
Spectrogram of recordings. (**a**) Cargo recording; (**b**) Passenger ship recording; (**c**) Tanker recording; (**d**) Environment noise recording.

The classification performance of the proposed model is verified on three ship types (Cargo, Passenger ship, Tanker) and Ocean environment noise. Each recording is a 5 min audio file in WAV format. The recordings are divided into training dataset and testing dataset, and each recording is splitted into segments of 6 s, which are considered as acoustic events. Data are normalized on each segment. Training samples and testing samples are 400 ms hopped 40 ms. The network training and testing is on raw time domain data without any preprocessing. The number of ships, number of acoustic events, total time of signals and number of samples are shown in Table 2. Spectrograms of recordings for the four categories are shown in Figure 2.

## 5. Experiments and Discussion

### 5.1. Experimental Setup

The proposed model is trained on raw time domain ship-radiated noise data. The performance of the proposed model is evaluated on three aspects:

1. The ship-radiated noise frequency decompose performance of the proposed model is observed by visualizing the outputs of filters in the deep filter sub-networks.
2. The classification performances of deep features extracted in the fusion layers, Layer-1 and Layer-2 in Figure 1, are observed by feature visualization method t-distributed stochastic neighbor embedding (t-SNE) [25].
3. The classification performance of the proposed model is evaluated by receivers operating characteristic (ROC) curve, area under ROC curves (AUC) value and classification accuracy, and is compared with several different methods.

The compared methods are as follows:

1. A DNN model trained on mel-frequency cepstral coefficients (MFCC) [26] features;
2. A DNN model trained on frequency spectrums that are calculated by short-term Fourier transform;
3. A CNN model trained on raw time domain signals [27].

### 5.2. Time Domain Ship Radiated Noise Decomposition

**Figure 3:**
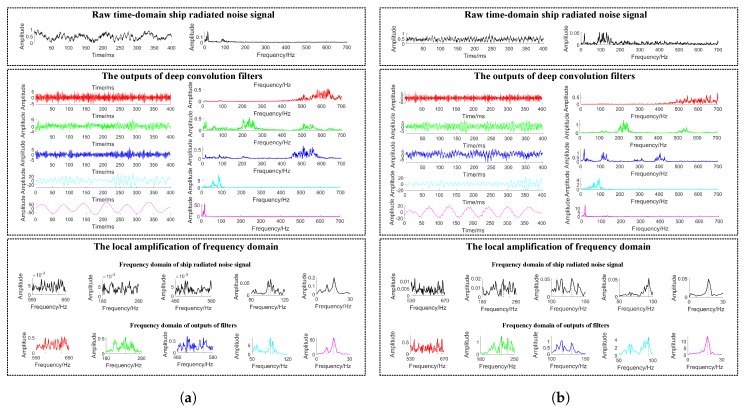
Visualization of the output of each filter. (**a**) Testing sample of Cargo class; (**b**) Testing sample of Passenger ship class.

Initialized the deep convolution filters randomly and then driven by the ship-radiated noise, the deep convolution filters are optimized for underwater acoustic target recognition. To verify whether these filters in the proposed model could learn the intrinsic properties of ship-radiated noise, the output of each learned deep convolution filter is visualized in both time domain and frequency domain. One selected testing sample is passed to the proposed model, and one of the outputs of each filter is extracted. The results are shown in Figure 3. Time domain of input signal and each output are shown in the left panel, frequency domain of input signal and each output are shown in the right panel. The local amplifications of frequency domain are shown on the bottom. As shown in Figure 3, the raw time domain ship-radiated noise as input signal and its frequency domain are plotted in black. The red line, the green line, the blue line, the cyan line and the magenta line are the outputs of filters in both time domain and frequency domain whose convolution kernels sizes get larger in turn. Filters with narrower convolution kernels have higher frequency and filters with wider convolution kernels have lower frequency generally. As described in Section 3, an auditory system is organized according to frequency. The proposed model could roughly match the property of the auditory system.

### 5.3. Feature Visualization by t-SNE

**Figure 4:**
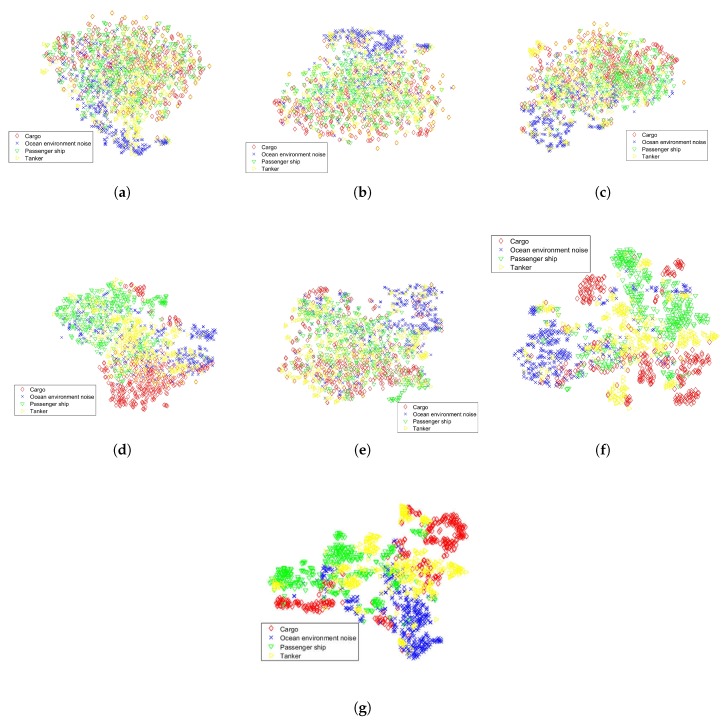
Result of t-SNE feature visualization. (**a**–**e**) Feature groups of deep filter sub-networks; (**f**) Features of layer-1; (**g**) Features of layer-2.

The cluster performance of the proposed method is visualized by t-SNE. 1400 testing samples randomly selected are used to plot the scatter diagram. The outputs of sub-networks and fusion layers are extracted as features. The re are five feature groups for five sub-networks respectively. The t-SNE is performed on each of the feature groups. Besides, the outputs of fusion layers, layer-1 and layer-2, are also visualized by t-SNE. The results are shown in Figure 4. Figure 4a–e are feature groups of sub-networks whose convolution kernels sizes get larger in turn. And Figure 4f,g are the features of layer-1 and layer-2 respectively. Samples from different classes are more overlapped in Figure 4a–e than in Figure 4f,g. By merging the five feature groups, the samples from different class are more distributed than each of the five feature groups, which indicated that the five groups are complementary. The layer-2 is the deepest layer in the proposed model, whose features are most discriminative.

### 5.4. Classification Experiments

To simulate the situation of practical application of ship-radiated noise recognition, classification performance of the proposed model is measured using the classification accuracy of each acoustic event, defined as the percentage of correctly classified acoustic events in all acoustic events. The accuracy of proposed model and the compared models are shown in Table 3.

**Figure 5:**
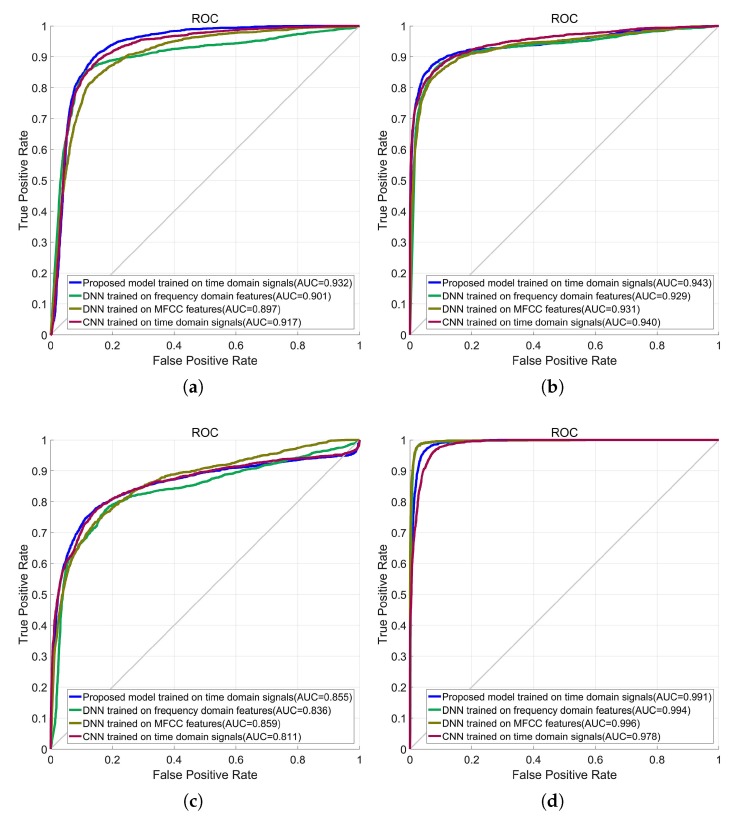
ROC curves of the proposed model and its competitors. (**a**) Cargo class is positive class; (**b**) Passenger ship class is positive class; (**c**) Tanker class is positive class; (**d**) Environment noise class is positive class.

ROC curves are plotted according to the classification results on testing data set. AUC values are plotted together with the ROC curves. Assuming that one class is positive class and others are negative class. The results are shown in Figure 5. As shown in Figure 5a,b, the proposed model has the highest AUC and in Figure 5c the proposed model has the second highest AUC. In Figure 5d, the AUC of proposed model is only 0.005 less than the highest.

Table 4 shows the confusion matrix of the proposed model obtained from testing data. The ship class with the best result is passenger ship class with classification accuracy of 87.08%. The poorest result is obtained for tanker class, the accuracy of which is 69.33%. This may be because the tanker has similar dynamical system and similar size with cargo. The ocean environment noise class has the best result with the classification accuracy of 94.17%. This indicates that it is easier for proposed model in detecting ships’ presence than classifying ship types.

## 6. Conclusions

In this paper, inspired by neural mechanisms of auditory perception, auditory perception inspired deep convolutional neural network is proposed for underwater acoustic target recognition. The deeper features with intrinsic information of target are extracted by each deep filter sub-network, which reflects the deep acoustic information extraction structure of auditory system. The ship-radiated noise signals are decomposed into different frequency components by the multi-scale deep convolution filters, which reflects the frequency component perception neural mechanism and reveals the distribution of different frequency component in the ship-radiated noise. Inspired by the plasticity neural mechanism, driven by the time domain ship-radiated noise, all of the parameters in ADCNN model are learned and optimized for underwater acoustic target recognition task. The experimental results show that, compared with compared methods, the proposed ADCNN achieves the best classification accuracy of 81.96% on the dataset including ocean environment noise and radiated noise from three ship types. The experimental results also show that auditory perception inspired deep learning methods have great potential to improve the classification performance of underwater acoustic target recognition.

## Figures and Tables

**Table 1:** The RMSProp algorithm.

The RMSProp algorithm
Input: $\theta_{1}\in R^{d},\delta >0,\alpha >0,v_{0}=0\in R^{d}$
For t=1 to *T* do
$g_{t}\in \partial f_{t}\left(\theta_{t}\right)$
$v_{t}=\beta_{t}v_{t-1}+\left(1-\beta_{t}\right)\left(g_{t}*g_{t}\right)$
Set $\epsilon_{t}=\frac{\delta }{\sqrt{t}}$ and $\alpha_{t}=\frac{\alpha }{\sqrt{t}}$
$A_{t}=diag\left(\sqrt{v_{t}}+\epsilon_{t}I\right)$
$\theta_{t+1}=P_{C}^{A_{t}}\left(\theta_{t}-\alpha_{t}A_{t}^{-1}g_{t}\right)$
End for

Where $\theta_{t}$ is the current parameter vector, *v* is the scaling vector, δ is a small constant, α is learning rate and β is step-size. *T* is the total iterations.

**Table 2:** Experimental data description.

Data Set	Class	No. Ships	No. Acoustic Event	Total Time (Hour)	No. Samples
Training	Cargo	13	6523	10.87	97,800
	Passenger ship	7	7326	12.21	109,900
	Tanker	35	5921	9.87	88,800
	Environment noise	non	10,497	17.49	157,400
Test	Cargo	9	1200	3.33	3000
	Passenger ship	10	1200	3.33	3000
	Tanker	16	1200	3.33	3000
	Environment noise	non	1200	3.33	3000

**Table 3:** Accuracy of different models.

Input	Methods	Accuracy/%
MFCC [26] features	DNN model	78.92
Frequency spectrum features	DNN model [8]	81.27
Raw time domain signal	CNN model [27]	77.01
Raw time domain signal	Proposed model	**81.96**

**Table 4:** Confusion matrix of the proposed model obtained from testing data. The bold numbers in diagonal indicate the number of correctly classified samples; the bottom right bold number indicates the overall accuracy.

	Predicted Label	Cargo	Passenger Ship	Tanker	Environment Noise	Recall (%)
Ture Label						
Cargo		**927**	100	141	32	77.25
Passenger ship		39	**1045**	80	36	87.08
Tanker		216	98	**832**	54	69.33
Environment noise		4	52	14	**1130**	94.17
Precision (%)		78.16	80.69	77.98	90.26	**81.96**
